# Prevention of Male Late-Onset Hypogonadism by Natural Polyphenolic Antioxidants

**DOI:** 10.3390/nu16121815

**Published:** 2024-06-09

**Authors:** Luc J. Martin, Mohamed Touaibia

**Affiliations:** 1 Biology Department, Université de Moncton, Moncton, NB E1A 3E9, Canada; 2 Chemistry and Biochemistry Department, Université de Moncton, Moncton, NB E1A 3E9, Canada; mohamed.touaibia@umoncton.ca

**Keywords:** androgen, testosterone, Leydig cells, testis, flavonoids, polyphenols

## Abstract

Androgen production primarily occurs in Leydig cells located in the interstitial compartment of the testis. In aging males, testosterone is crucial for maintaining muscle mass and strength, bone density, sexual function, metabolic health, energy levels, cognitive function, as well as overall well-being. As men age, testosterone production by Leydig cells of the testes begins to decline at a rate of approximately 1% per year starting from their 30s. This review highlights recent findings concerning the use of natural polyphenolics compounds, such as flavonoids, resveratrol, and phenolic acids, to enhance testosterone production, thereby preventing age-related degenerative conditions associated with testosterone insufficiency. Interestingly, most of the natural polyphenolic antioxidants having beneficial effects on testosterone production tend to enhance the expression of the steroidogenic acute regulatory protein (*Star*) gene in Leydig cells. The STAR protein facilitates the entry of the steroid precursor cholesterol inside mitochondria, a rate-limiting step for androgen biosynthesis. Natural polyphenolic compounds can also improve the activities of steroidogenic enzymes, hypothalamus-pituitary gland axis signaling, and testosterone bioavailability. Thus, many polyphenolic compounds such as luteolin, quercetin, resveratrol, ferulic acid phenethyl ester or gigantol may be promising in delaying the initiation of late-onset hypogonadism accompanying aging in males.

## 1. Introduction

Androgens are mainly produced by testis Leydig cells in vertebrates. These steroid hormones are essential for the development, as well as the maintenance, of male sex organs and secondary sexual characteristics. In the embryo, testosterone stimulates the development of Wolff’s ducts, which will differentiate into the epididymis, vas deferens and seminal vesicles. In addition, testosterone is converted into dihydrotestosterone (DHT) to stimulate the development of the penis, scrotum and prostate from the genital tubercle. At puberty, testosterone is responsible for masculinization of the central nervous system, initiation and maintenance of spermatogenesis, differentiation of external genitalia, hair growth, bone growth, and regulation of the secretion of gonadotropins luteinizing hormone (LH) and follicle-stimulating hormone (FSH). In the seminiferous tubules of the testis, androgens interact with the androgen-binding protein (ABP, SHBG) in Sertoli cells, supporting the division of germ cells and normal spermatogenesis. Male hypogonadism is characterized by lower-than-normal production of testosterone by the testes, which may result in decreased sperm production. From their thirties, testosterone production by Leydig cells in the testis declines at a rate of 1% per year in men [1]. This decline in serum testosterone levels, contributing to the development of late-onset hypogonadism, is accentuated by a variety of conditions, including exposure to endocrine disruptors, metabolic disorders, and cancer treatments [2]. With aging, Leydig cells respond less effectively to the pituitary hormone LH to stimulate androgen production [3]. Men with lower-than-normal serum levels of androgen may experience decreased libido, erectile dysfunction, reduced body hair, decreased muscle mass, low bone mineral density, increased body fat, increased fatigue, irritability, difficulty to concentrate, as well as poor overall quality of life [4,5]. Therefore, therapies aimed at restoring serum testosterone levels to normal, including nutritional supplementation with polyphenolic compounds, may alleviate some of these symptoms. In this review, the structure-activity relationship of plant-derived polyphenolic compounds on androgen production will be considered as a potential approach to limit the development of male late-onset hypogonadism.

The literature search methodology for this review consisted in searching for scientific publications found in the bibliographic databases NCBI-PubMed and Google Scholar using the following keywords: (Natural antioxidant OR Flavonoid OR Phenolic OR Polyphenol OR Polyphenolic) AND (Testosterone OR Androgen OR Leydig), with no limitations on date of publication. All relevant scientific works identified as suitable for this topic were included in this review. This literature review will focus on natural polyphenol antioxidants because of their potential to enhance the steroidogenic capacity of Leydig cells, as demonstrated by our laboratory research [6,7,8,9,10]. In addition, polyphenolic compounds have anti-aging properties such as the capacity to inhibit apoptosis caused by reactive oxygen species, and the ability to increase the activity of oxidative stress-limiting enzymes like superoxide dismutase, glutathione peroxidase and catalase [11,12]. Moreover, flavonoids such as quercetin may prevent aging-related diseases by targeting the NAD+-dependent deacetylase enzyme sirtuin involved in the regulation of cellular senescence and multiple aging-related cellular processes [13]. In Leydig cells, sirtuin may serve as an interesting target to restore mitochondrial dysfunction and enhance steroid production.

## 2. Testosterone Synthesis by Leydig Cells

**Figure 1:**
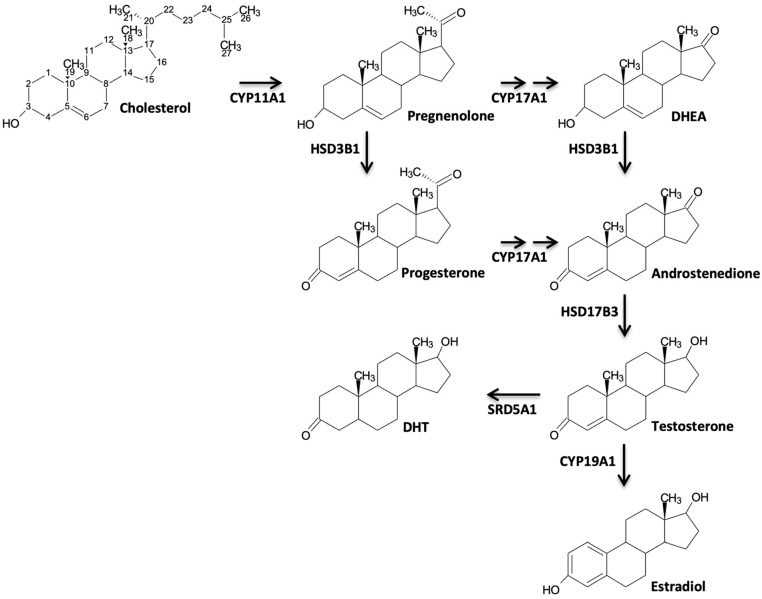
Common synthesis pathway for testosterone from cholesterol and its subsequent conversion into estrogenic and androgenic active metabolites. Abbreviations for enzymes: CYP11A1, P450 side chain cleavage; HSD3B1, 3β-hydroxysteroid dehydrogenase; CYP17A1, P450 17α-hydroxylase/20-lyase; HSD17B3, 17β-hydroxysteroid dehydrogenase; SRD5A1, 5α-reductase; CYP19A1, P450 aromatase. Abbreviations for steroids: DHEA, dehydroepiandrosterone; DHT, dihydrotestosterone.

In the testis, the biosynthesis of testosterone occurs in Leydig cells. Androstenedione and dehydroepiandrosterone (DHEA) are also produced; however, their efficacy to activate the androgen receptor is lower than testosterone. Adult Leydig cells also contain the aromatase enzyme (CYP19A1) involved in the transformation of androgen into estrogen [14]. However, such rate of conversion of testosterone into estradiol is relatively minimal, and estrogen may rather moderate steroid production by Leydig cells [15]. The biosynthesis of testosterone relies on the steroidogenic enzymes cholesterol side chain cleavage enzyme (CYP11A1), cytochrome P450 17α-hydroxylase/20-lyase (CYP17A1), 3β-hydroxysteroid dehydrogenase (HSD3B2 in humans and HSD3B1 in rodents) and 17β-hydroxysteroid dehydrogenase type 3 (HSD17B3) with cholesterol as the initial substrate (Figure 1). Cholesterol can be produced from acetyl CoA or obtained from plasma using receptor mediated endocytosis of LDL particles. Cholesterol can be stored as an esterified form in lipid droplets in the cytoplasm of Leydig cells. These cells depend primarily on endogenous cholesterol synthesis for testosterone biosynthesis under normal physiological conditions. However, the increased demand for cholesterol can be satisfied by increased uptake of extracellular cholesterol from LDL and HDL via endocytosis mediated by the surface receptors LDLR and scavenger receptor class B, type I (SCARB1) [16].

The initial reaction in steroid production requires the translocation of cytoplasmic cholesterol inside mitochondria. This is initiated by the assembly of a carrier protein complex, including the steroidogenic acute regulatory protein (STAR) and the translocator protein (TSPO), at the outer mitochondrial membrane [17,18]. Inside mitochondria, cholesterol is metabolized to pregnenolone by the CYP11A1 enzyme with the implication of the electron carrier ferredoxin and the electron donor NADPH: ferredoxin reductase [19]. Once produced, pregnenolone leaves the mitochondria and enters the smooth endoplasmic reticulum (SER) by diffusion to complete testosterone synthesis with the HSD3B, CYP17A1 and HSD17B3 enzymes. Testosterone is a critical endocrine and paracrine steroid hormone initiating and maintaining spermatogenesis in the testes, and male secondary sexual characteristics. During aging, STAR protein levels are reduced in adult Leydig cells, leading to a reduction in mitochondrial cholesterol import and a decline in androgen production [20,21]. This age-related decline in the production of testosterone can be delayed by maintaining expression of the *Star* and/or *Cyp11a1* genes with the aid of polyphenolic antioxidant supplementation [22,23].

The hormone LH activates the expression of genes encoding steroidogenic enzymes via activation of the cAMP/protein kinase A (PKA) signaling pathway in Leydig cells. PKA substrates include STAR, responsible for cholesterol transport within the mitochondria, and several transcription factors essential for regulating steroidogenic gene expression. In addition to the cAMP/PKA pathway, other signaling pathways such as mitogen activated protein kinases (MAPK), protein kinase C (PKC), Ca^2+^-calmodulin dependent protein kinases (CAMK) and Janus kinases/signal transducer and activator of transcription proteins (JAK/STAT) regulate steroidogenesis in testis Leydig cells. Indeed, activation of the epidermal growth factor receptor (EGFR) regulates steroidogenesis by modulating the activity of the extracellular signal-regulated kinases (ERK1/2) component of MAPK signaling [24,25]. The adipose derived hormones leptin and resistin regulate steroidogenesis through the activation of JAK/STAT pathway in Leydig cells [26,27]. Increased intracellular Ca^2+^ levels lead to accumulation of calmodulin-associated Ca^2+^ and activation of Ca^2+^/calmodulin kinase kinase (CAMKK). The latter specifically phosphorylates and activates Ca^2+^/calmodulin kinase I (CAMKI) in adult Leydig cells, playing an important role in regulating *Star* gene expression [28]. Activation of both PKC and PKA contributes to the upregulation of STAR and steroidogenesis in Leydig cells [29,30]. Nutrients, such as plant polyphenolic compounds, can promote testosterone production in Leydig cells via various regulatory mechanisms, including modulation of intracellular signal transduction pathways, and these actions may depend on a specific structure-function relationship.

## 3. Development of Late-Onset Male Hypogonadism

Late-onset male hypogonadism is characterized by a decline in testosterone production in the testes as a function of aging. This condition is usually treated with testosterone replacement therapy, leading to undesirable side effects. For instance, spermatogenesis is dramatically reduced, leading to decreased fertility [31]. This results from negative feedback by exogenous testosterone on the hypothalamus and pituitary, resulting in decreased secretion of the gonadotropin releasing hormone (GnRH), as well as of FSH and LH.

Testosterone plays several important roles in aging males by maintaining muscle mass and strength, bone density, sexual function, energy level, metabolic health, cognitive function, and overall well-being (Table 1). As men age and testosterone levels decline, they may experience a gradual loss of muscle mass and strength, known as sarcopenia [32]. Low testosterone levels in aging males can lead to a decrease in bone mineral density, increasing the risk of osteoporosis and fractures [33]. Declining testosterone levels can result in reduced sexual desire, erectile dysfunction, and other sexual problems [34]. Low testosterone levels in aging males may contribute to feelings of fatigue, decreased motivation, and reduced energy levels [35]. Testosterone has an impact on metabolism, including the regulation of fat distribution, insulin sensitivity, and glucose metabolism (Table 1) [36]. Hence, low testosterone levels may contribute to an increased risk of obesity, metabolic syndrome, and type 2 diabetes in aging males [37]. Although not clearly established, some research suggests that testosterone may have a positive effect on cognitive function, such as memory and concentration [38,39]. Hence, low testosterone levels have been associated with cognitive decline [40], increased risk of Alzheimer’s disease [41], and symptoms of depression [42].

Thus, maintaining adequate testosterone levels is important for various aspects of health and well-being in aging males. Natural antioxidants such as polyphenolic compounds from fruits and vegetables may promote optimal testosterone production and delay symptoms associated with late-onset hypogonadism in aging males. 

## 4. Natural Antioxidants Contributing to the Optimal Production of Androgen

### 4.1. Flavonoids

**Figure 2:**
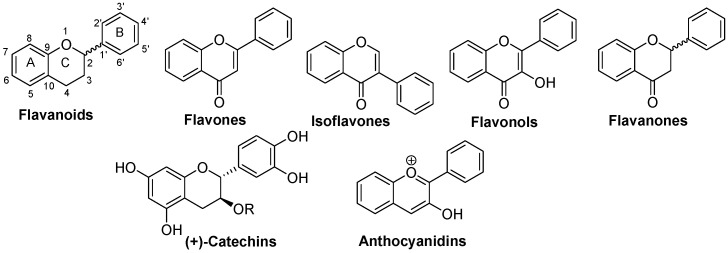
Structures of important flavonoids found in plants.

Flavonoids are of vital and crucial importance to many plants, and they are found in various parts of the plant. These constituents play an important role in the development of plants, as well as in their defense against pathogens [66]. Flavonoids’ basic skeleton is characterized by two benzene rings linked by a three-carbon segment. The different types of flavonoids can be distinguished by the oxygenated heterocycle (ring C) created by the three-carbon segment, its oxidation level, and the hydroxyl groups (-OH) of ring A (see Figure 2 for the structures of important flavonoids found in plants).

Flavonoids can be divided into flavanone, flavones, flavonols and anthocyanidins, based on distinctions in the 2-phenyl-3,4-dihydro-2H-1-benzopyran backbone [67]. Isoflavonoids can be classified in two main families: isoflavans and isoflavones, both with a 3-phenyl-3,4-dihydro-2H-1-benzopyran backbone [67]. The presence of oxygen, either with hydroxyls or ketones, reduces the lipophilicity of the molecules while increasing the possibility of hydrogen bonds formation (Table 2). The biological effect of these molecules may be partly due to their solubility, but largely to the presence of specific receptors or favorable interactions between the molecule and its target. Prenylated flavonoids are an excellent example of increased lipophilicity leading to greater affinity with the cell membranes [68].

Since they were first characterized, flavonoids have been associated with a number of health benefits, including cancer prevention, reducing the risk of cardiovascular and neurodegenerative diseases, and delaying age-related symptoms (reviewed in [70]). Since the early 60s, over 900 publications have reported on the actions of various flavonoids on androgen production. However, the molecular mechanisms by which flavonoids regulate steroid biosynthesis remain to be better defined.

#### 4.1.1. Flavones

Flavones are characterized by the presence of a ketone moiety and a double bond in the ring C (Figure 1). Luteolin and apigenin are flavones found in celery, thyme and parsley. Interestingly, we found that chrysin, apigenin, luteolin and baicalein can stimulate the cAMP-dependent expression of *Star*, *Cyp11a1* and *Fdx1* (Ferredoxin 1) in MA-10 Leydig cells [7]. However, only luteolin is able to increase the cAMP-dependent progesterone synthesis in MA-10 Leydig cells. In addition, luteolin also activates *Star* expression and increases the productions of progesterone and testosterone in LC540 tumor Leydig cells [10]. Apigenin and chrysin have also been reported by others to increase cAMP-dependent androgen production from testis Leydig cells by enhancing the expression of the *Star* gene [71,72].

Chrysin and apigenin can reduce the protein levels of DAX1 (dosage-sensitive sex reversal, adrenal hypoplasia critical region, on the X chromosome, gene 1), a major inhibitor of *Star* expression [72]. In Leydig cells from aging males, COX2 (cyclooxygenase-2) is associated with reduced expression of *Star*, leading to lower production of testosterone [73]. Interestingly, apigenin can inhibit *Cox2* expression, leading to increased *Star* expression [72]. However, apigenin can also inhibit the production of 5α-androstane-3α, 17β-diol (DIOL), the major androgen produced by immature rat Leydig cells, as well as the steroidogenic enzymes HSD3B, CYP17A1 and HSD17B3 [74]. In addition, human HSD3B2, CYP17A1, and HSD17B3 were more sensitive to the inhibitory action of apigenin, decreasing androstenedione and testosterone productions in H295R human adrenal cells [75,76]. Overall, apigenin contributes to increased steroid production in Leydig cells by increasing PKA-dependent STAR protein levels. However, this effect may not result in improved testosterone synthesis, as apigenin also inhibits the expression of *Cyp11a1* in LC540 tumor Leydig cells [10].

We have reported that luteolin can improve androgen production from Leydig cell by increasing *Star* expression [7,10]. Indeed, luteolin can increase cAMP-dependent steroid production by increasing STAR protein levels and the import of cholesterol inside mitochondria in MA-10 Leydig cells [77]. Luteolin can also increase *Fdx1* expression in MA-10 Leydig cells [7]. FDX1 is involved in the electron transfer to support the activity of the steroidogenic enzyme CYP11A1. As for apigenin and chrysin, luteolin also inhibits *Cox2* expression, thus improving *Star* transcription [78]. As reported for other flavonoids, luteolin also increases *Star* expression by reducing the expression of *Dax1* [77].

The 5,7-dihydroxyflavone chrysin can be found in honey, chamomile, propolis, fruit bark as well as many plant extracts. In MA-10 Leydig cells, treatment with chrysin can stimulate cAMP-dependent androgen production by increasing STAR protein levels and steroidogenesis, possibly by decreasing *Dax1* expression [71]. Chrysin can also inhibit the activity of NF-κB transcription factors, resulting in decreased activity of the *COX2* promoter [79]. Reduced levels of COX2 in Leydig cells may promote *Star* expression. Chrysin is also an inhibitor of the aromatase enzyme, responsible for the conversion of testosterone into estradiol [80]. Such inhibitory action of flavonoids on the aromatase enzyme has also been attributed to apigenin [81]. Overall, chrysin can increase the serum levels of testosterone by more than 35% in adult male rats [82]. However, treatment with chrysin for 21 days had no effect on the urine concentration of testosterone in humans [83]. This may be explained by the low concentration of chrysin utilized in this study compared to those on rodents. However, treatment of male mice for 10 days with 20 mg/kg of chrysin prevents the inhibition of testosterone production following exposure to the mycotoxin zearalenone, but does not affect serum testosterone levels [84]. Therefore, chrysin may delay the age-related decline in STAR expression and testosterone production by Leydig cells [71,84,85]. However, chrysin did not induce an increase in steroid production when co-incubated with 22(R)hydroxycholesterol in MA-10 Leydig cells [71]. Thus, chrysin only enhances cholesterol entry into mitochondria via STAR and has no effect on the activities of enzymes involved in steroidogenesis. Recently, chrysin has been reported to improve recovery from heat stress, resulting in improved testosterone production from rat Leydig cells [86]. This flavone also protects against testicular apoptosis induced by torsion/detorsion and improves sperm quality in adult rat [87].

Baicalein can be found in several plants such as roots of *Oroxylum indicum* [88] and *Scutellaria baicalensis* Georgi [89]. As for other flavones, low concentrations of baicalein can improve cAMP-dependent activation of the *Star* promoter in MA-10 Leydig cells [7]. In addition, the activities of the *Cyp11a1* and *Fdx1* promoters are also increased by co-treatments with cAMP as observed with the other flavones luteolin, apigenin and chrysin. However, baicalein administration for 4 weeks has been reported to reduce serum levels of testosterone, FSH and LH following treatments of a rat polycystic ovary syndrome (PCOS) model [90]. In this study, baicalein rather decreases *Star*, *Cyp11a1*, *Hsd3b*, and *Cyp19a1* expressions in ovarian tissues. Such a variation in responses to baicalein may be due to the sex of the animals and to the pathological state, such as PCOS. Overall, flavones appear to have an activating effect on the expression of steroidogenesis-related genes, including *Star*, in Leydig cells. However, these effects do not necessarily lead to an increase in androgen production.

#### 4.1.2. Isoflavones

Isoflavones, such as genistein and daidzein, can be found in soybeans, red clover, alfalfa, and chickpeas. In mice, all testicular cell types express the estrogen receptor (ER)-β, while Leydig cells predominantly express ER-α [91]. In the testis, the aromatase enzyme is found mainly in Sertoli cells, enabling them to synthesize estrogen from androgen [92] and leading to ER-α-mediated inhibition of steroidogenesis in Leydig cells [93]. However, as adult Leydig cells produce increasingly higher levels of estrogen [94], the importance of this autocrine action on steroid production by Leydig cells remains to be better defined. With their phytoestrogenic effects, isoflavones can disrupt paracrine and autocrine estrogen signaling in the testis. Isoflavones have direct effects on the steroidogenic function of Leydig cells. Indeed, daidzein inhibits testosterone production by Leydig cells by decreasing *Star*, *Cyp11a1* and *Hsd3b1* expression levels in the testes of neonatal mice [95]. Genistein inhibits progesterone production by decreasing *Star* expression in MA-10 Leydig cells [96]. Furthermore, genistein also inhibits testosterone production in fetal mouse testes by decreasing expression levels of *Star*, *Cyp11a1*, *Hsd3b1* and *Cyp17a1* [97]. HSD3B and HSD17B3 enzyme activities are also inhibited by genistein in rat and human testes [98]. Thus, high concentrations of isoflavones may reduce steroidogenesis in the testis.

The different effects of flavonoids and isoflavones on androgen production can be explained by a structure-activity relationship. Indeed, isoflavones with the phenol group in position 3 of the C ring (Figure 1), such as daidzein and genistein, tend to inhibit the activities of the HSD3B2 and HSD17B3 enzymes, rather than aromatase [99]. This could explain the greater capacity of inhibition by isoflavones on testosterone production in H295R adrenal cells, compared to a flavonoid such as apigenin, whose phenolic group is in position 2 of the C ring [75].

Although several studies suggest that isoflavones contribute to lower testosterone levels in men, a meta-analysis of 32 studies concluded that neither soy foods nor isoflavone supplementation had significant effects on testosterone levels [100]. Furthermore, treatment of MA-10 Leydig cells with up to 100 μM genistein for 48 h has no effect on cell viability, progesterone synthesis and expression of steroidogenesis-related genes *Star*, *Tspo*, *Cyp11a1* and *Hsd3b1* [101]. However, Leydig cell proliferation and increased levels of STAR, CYP11A1, HSD3B and CYP17A1 are observed following exposure of perinatal male rats to soy isoflavones [102]. In contrast, exposure to genistein during perinatal development of male rodents results in reduced anogenital distance, decreased testicular mass and lower serum testosterone levels [103,104,105]. Specifically, a low concentration of 10 nM genistein inhibits testosterone production by fetal Leydig cells through interaction with the estrogen receptor α and decreases expression of the *Star* gene and of enzymes related to steroidogenesis [97]. Thus, exposure of the male fetus to phytoestrogens may disrupt testicular development and function.

The enzyme 5-α-reductase catalyzes the synthesis of 5-α-dihydrotestosterone (DHT) and is inhibited by genistein and daidzein in vitro [106], contributing to the reduction in serum levels of DHT in male rats [107]. This steroid can contribute to the development of prostate cancer [108]. While short-term treatment with genistein for 5 days result in a modest increase by less than 10% of plasma testosterone levels in Wistar rats [109], some have reported that a lifetime exposure to daidzein and genistein increases serum and testicular testosterone levels [110]. Others have shown that treatments with soy isoflavones daily for 8 weeks partially prevents the decrease in testosterone serum levels associated with aging in male rats [111]. However, a long-term consumption of daidzein and genistein impairs normal androgen production and reproductive function in male Wistar rats [112]. Hence, the effects of prolonged exposures to isoflavones on testosterone production may depend on the concentration, duration, as well as ratio of daidzein to genistein.

Interestingly, effects of genistein exposure of male pups from two weeks before birth until PND35 may be dependent on the concentration. Indeed, a low concentration of genistein (40 mg/kg) results in increased body weight, testosterone serum levels, testis weight and size of seminiferous epithelium, as well as increased expression of *Esr2*, *Sox9*, *Brd7* and *Cyp19a1* [113]. Oppositely, exposure to a high concentration of genistein (800 mg/kg) results in increased numbers of abnormal spermatids, higher apoptosis of germ cells, decreased diameter of seminiferous tubules, body and testis weights, whereas the expressions of *Esr2* and *Cyp19a1* were increased and the expression of *Sox9* and *Brd7* were decreased [113]. However, high concentration of genistein (800 mg/kg) has no effect on testosterone serum levels according to this study. Others have reported that soybean meal and genistin/daidzin diets decrease testosterone production regardless of the age of male rats due to decreases in STAR and HSD17B protein levels [114]. Genistin and daidzin are 7-O-β-D-glucosides of genistein and daidzein, respectively, and are metabolized into their aglycones during intestinal absorption. In addition, genistein and daidzein decrease LH stimulated testicular and Leydig cells’ testosterone production [114], suggesting that isoflavones mainly inhibit Leydig cells’ function rather than disrupting the entire hypothalamus–pituitary–gonadal (HPG) axis. Importantly, in a meta-analysis of clinical studies published between 2010 and 2022, no significant effects of soy isoflavones intake, regardless of dose and study duration, were found on testosterone levels in men [115].

#### 4.1.3. Flavonols

With their ketone moiety, flavonols are the most oxidized flavonoids (Figure 1). Quercetin, myricetin and kaempferol are flavonols found in berries, apples, onions, broccoli, and tea. Flavonols have been well documented for their ability to enhance steroidogenesis and testicular function. Indeed, quercetin, myricetin or pentaacetylquercetin increase the cAMP-dependent expression of *Star*, *Cyp11a1* and *Fdx1*, thus contributing to increased steroidogenesis in MA-10 Leydig cells [6]. However, quercetin supplementation of healthy men for 8 weeks has no effect on their serum testosterone levels [116]. This discrepancy in the effects of quercetin on testosterone synthesis can be explained by differences in its mechanism of action between species.

Quercetin improves steroid synthesis and testosterone levels in male mice exposed to the endocrine disruptor bisphenol A [117]. Indeed, quercetin increases the transcriptional activity of CREB1, as well as the promoter activities of *Cyp11a1* and *Fdx1* [6]. The CREB1 transcription factor is an important activator of steroidogenic genes, including *Star*, in Leydig cells [118,119,120]. Thus, quercetin increases *Star* promoter activity, *Star* mRNA levels and steroid production in MA-10 Leydig cells [96]. This quercetin-dependent increase in the expression of the *Star* gene in response to cAMP can also be attributed to the decrease in DAX1 protein levels in Leydig cells [77]. In addition, *Star* expression and steroidogenesis may be enhanced by inhibition of COX2 signaling following treatment with quercetin, as observed with chrysin, apigenin or luteolin in Leydig cells [77]. The activity of COX2 increases in aged Leydig cells, leading to repression of *Star* gene expression and androgen production [73]. Hence, consumption of polyphenolic antioxidants, including flavonoids, may delay the decline in age-related androgen production in men. Quercetin can also inhibit endoplasmic reticulum stress and improve testosterone production in streptozotocin-induced diabetic male rats and high glucose-treated Leydig cells [121]. Moreover, quercetin can enhance testosterone synthesis in rats exposed to cadmium chloride or the herbicide atrazine by improving the activities of the HSD3B and HSD17B3 enzymes [122,123]. We have also confirmed that quercetin can increase *Hsd3b* expression in rat LC540 Leydig cells [10]. In addition, quercetin-derived molecules such as pentaacetylquercetin can activate *Star* and *Cyp11a1* expression, leading to an increase in cAMP-dependent progesterone accumulation in MA-10 Leydig cells [6]. This acetylation of quercetin may improve its bioavailability in vivo.

Icariin, a prenylated flavonol glycoside, has been found in several plant species of the genus *Epimedium*, including horny goat weed. Icariin can reverse the negative effects of di(2-ethylhexyl) phthalate (DEHP) on the proliferation and testosterone synthesis capacities of primary Leydig cells [124]. Indeed, icariin prevents DEHP-induced decreases in the expression of steroidogenic enzymes (CYP11A1, HSD3B1 and HSD17B3) and of the transcription factor NR5A1 (SF-1), an important regulator of the expression of steroidogenic genes [125,126]. In addition, icariin increases the expression of TSPO and STAR, facilitating cholesterol entry into mitochondria and promoting testosterone production in adult male rat testes [127]. Icariin also improves the expression of *Star* and *Hsd3b1*, as well as of genes encoding superoxide dismutase (*Sod1*, *Sod3*) and glutathione peroxidase (*Gpx1*), leading to increased serum levels of testosterone after 35 days of treatment of male mice [128]. However, icariin also activates the apoptosis of mouse Leydig tumor cells (mLTC1) [129].

Rutin is a glycosylated quercetin commonly found in citrus fruits. It has been shown to reduce the decline in serum LH, FSH and testosterone levels, as well as improving sperm quality, following exposure of male rats to carbon tetrachloride, a major environmental contaminant [130]. Rutin also reduces the decrease in serum testosterone levels in response to cadmium by increasing the activities of HSD3B and HSD17B3 enzymes in male rats [131,132]. In addition, rutin can improve the expressions of *Cyp11a1* and *Hsd3b1*, but with no effect on serum testosterone levels in male rats [133]. However, others reported that rutin rather inhibits basal and LH-induced secretions of androgen by rat immature Leydig cells by decreasing the expressions of *Scarb1*, *Cyp11a1* and *Hsd3b1* [134].

Taxifolin or dihydroquercetin belongs to the flavanonol subclass and is found in red onions. This molecule inhibits the enzymes HSD3B and CYP17A1, leading to a reduction in androgen production in immature rat Leydig cells [135]. In contrast, human HSD3B2 and CYP17A1 enzymes are less sensitive to taxifolin. Overall, flavonols are natural antioxidants that can enhance the activities of steroidogenic enzyme and prevent age-related declines in androgen production in men.

#### 4.1.4. Flavanones

Flavanones lack a double bond in their C ring (Figure 2). The flavanone naringenin, found in grapefruits, inhibits the activities of HSD17B3 and HSD3B enzymes in male rat testes [136]. However, treatments of male rats with naringenin for 10 weeks results in increased serum levels of testosterone [137]. Hence, the conditions of flavonoid treatments, such as concentration, duration and route of administration, can influence the results in certain experimental models. Naringenin also prevents the decrease in serum testosterone and inhibin B in rats treated with chemotherapeutic agents such as cisplatin and doxorubicin [138]. In addition, cadmium-induced testicular toxicity can be reduced by treatment of male rats with naringenin, resulting in partial recoveries of GnRH, FSH, LH and testosterone serum levels [139]. Naringin, a flavanone-7-O-glycoside derived from naringenin, is also able to improve testosterone production in response to treatments with bisphenol A or high fat diet-induced diabetes in male rats [140,141]. Hesperetin, a flavanone found in citrus fruits, can also prevent reduced testosterone production in diabetic rats [142]. In addition, hesperidin, a glycosylated form of hesperetin, can prevent the decline in testosterone serum levels resulting from exposure to DEHP of male Wistar rats [143]. Hesperidin glycoside, also found in citrus fruits, prevents a decrease in serum testosterone levels in male rats treated with vanadium [144]. Thus, flavanones are promising natural antioxidants for limiting the decline in androgen levels in response to endocrine disruptors and possibly aging.

#### 4.1.5. Catechins

Catechins are distinguished by the lack of a double bond in the C ring and by the presence of an -OH group in position-3 of this ring (Figure 2). With dihydrochalcones, catechins are the most reduced flavonoids. Catechins are mainly found in apples, red wine and tea. Catechin, epicatechin and epigallocatechin gallate (EGCG) are able to increase plasma levels of testosterone in male rats [145]. Indeed, catechins increase testosterone production in response to hCG in rat Leydig cells [145]. In addition, epicatechin increases the activity of the HSD17B3 enzyme. However, others have shown that green tea polyphenols rather inhibit androgen synthesis in rat Leydig cells via inhibition of the PKA/PKC signaling pathways, and of the CYP11A1 and HSD17B3 enzymes [146]. Furthermore, long-term consumption of green tea is associated with an increase in aromatase expression, leading to a decrease in plasma levels of testosterone in humans [147]. In contrast, catechin inhibits aromatase activity, leading to an increase in plasma testosterone levels in male rats [148]. Furthermore, catechin injections also contribute to increasing plasma testosterone levels [145]. Green tea EGCG inhibits the productions of progesterone and estradiol in porcine granulosa cells [149]. In contrast, EGCG also inhibits the activities of the PKA/PKC signaling pathways, and the enzymes CYP11A1 and HSD17B3, leading to a reduction in testosterone production in primary Leydig cells [146]. Overall, these contradictory effects of catechins on plasma testosterone levels require further research to better define their mechanisms of action and could be attributed to different actions of catechins depending on the species.

#### 4.1.6. Anthocyanidins

Anthocyanidins are characterized by a positive charge and two double bonds on the C ring (Figure 2). Berries, currants, grapes, tropical fruits, as well as wine and tea, are rich in these colored pigments. Anthocyanidins are known for their antioxidant and antimicrobial capacities [150]. These flavonoids may improve steroidogenesis through their inhibitory activity against COX2 and their capacities to modulate the activity of the MAPK signaling pathway [151,152], promoting STAR protein expression and activity in Leydig cells. Cyanidin-3-glucoside prevents lead (Pb)-induced inhibition of progesterone production in R2C Leydig cells by preserving the integrity of mitochondria and increasing the expression of steroidogenic genes *Star*, *Hsd3b* and *Cyp11a1* [153]. This anthocyanin also activates the MAPK and PKA signaling pathways [153], thus promoting steroid synthesis. Cyanidin-3-glucoside also enhances testicular expression of the steroidogenic proteins STAR, CYP11A1 and HSD3B, as well as the LH receptor, in mice exposed to cadmium, a major neuro-endocrine disruptor [154]. Therefore, anthocyanidins may enhance testosterone production by Leydig cells via their antioxidant capacities.

### 4.2. Hydroxycinnamic Acid Phenethyl Ester Derivatives

**Figure 3:**
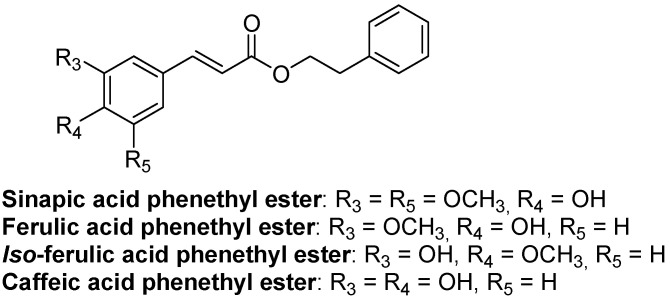
Structures of sinapic, ferulic, iso-ferulic, and caffeic acid phenethyl esters.

Present in many plants, hydroxycinnamic acids are biosynthesized via the shikimic pathway with L-tyrosine or L-phenylalanine, via methylation, deamination, or hydroxylation reactions [155]. We recently reported that treatments with 10 μM of sinapic or ferulic acid phenethyl esters (Figure 3) increase progesterone production in MA-10 Leydig cells [9]. Moreover, genes encoding enzymes contributing to cholesterol and steroid biosynthesis are increased by sinapic acid phenethyl ester, whereas ferulic acid phenethyl ester increases cAMP-dependent STAR protein expression. In addition, others have reported that caffeic acid phenethyl ester (Figure 3) reduces cadmium-induced apoptosis of Leydig cells and disruption of the production of testosterone [156]. Thus, these studies suggest that methoxycinnamic acid phenethyl ester derivatives can enhance androgen production by Leydig cells by improving the expression of steroidogenesis-related genes.

Also having hydroxy-methoxyphenyl groups, curcumin rather decreases LH-stimulated testosterone production by inhibiting the HSD17B1 enzyme in adult rat Leydig cells [157]. In addition, curcumin inhibits cAMP-induced steroidogenesis by inhibiting the levels of STAR and CYP11A1 in mouse Leydig cells [158].

### 4.3. Resveratrol and Gigantol

**Figure 4:**
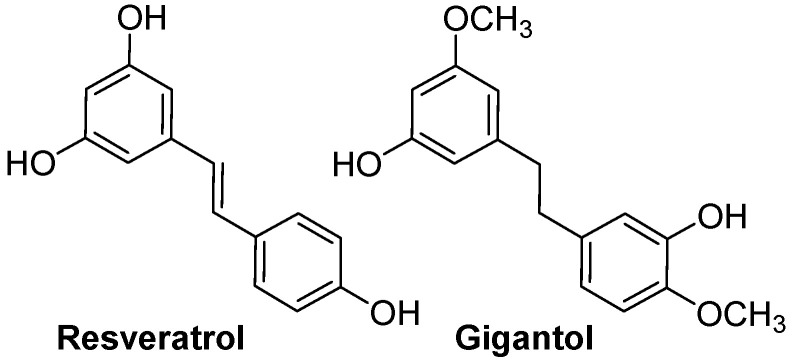
Structures of resveratrol and gigantol.

Resveratrol (3,4′,5-trihydroxy-trans-stilbene, Figure 4) is a polyphenol found in grapes, berries, peanuts, plums and red wine. As for flavonoids, it is essential for protecting plants from environmental stress and pathogen invasions [159]. Because of its structural similarity with estradiol, resveratrol is qualified as a phytoestrogen [160].

Numerous studies have reported that resveratrol enhances spermatogenesis, testosterone production and sperm quality [161,162]. However, others have reported that resveratrol disrupts LH-stimulated androgen production by Leydig cells by inhibiting the HSD3B1 enzyme in immature rats [163]. In addition, resveratrol can also inhibit the expression of STAR and CYP17A1 in rat Leydig cells [164], as well as *Star* promoter activity in mouse MA-10 Leydig cells [96]. Inhibition of androgen production by resveratrol may also involve its capacity to activate the adenosine monophosphate-activated protein kinase (AMPK) [165], known to inhibit the expression of important regulators of steroidogenesis, such as the transcription factor NR4A1 and STAR, in Leydig cells [166]. On the other hand, others suggest that resveratrol is promising for the treatment of male obesity-associated secondary hypogonadism. Indeed, resveratrol prevents the decrease in the expressions of *Star*, *Cyp11a1*, *Cyp17a1* and *Hsd17b3* and restores testosterone serum levels in response to a high-caloric diet in mice [167]. In addition, resveratrol can also upregulate mitochondrial biogenesis and steroidogenesis in aged Leydig cells [168], potentially reducing the symptoms of late-onset male hypogonadism. Furthermore, aging Leydig cells are exposed to increasing levels of oxidative stress, and resveratrol can prevent the detrimental effects of such stress on mitochondrial function and steroidogenesis, as reported in TM3 Leydig cells [169,170]. Others have also reported that resveratrol can reduce nicotine-induced oxidative damage in TM3 Leydig cells by upregulating autophagy through activation of the AMPK pathway [170].

Interestingly, a microgreen extract from cress (*Lepidium sativum*) rich in ferulic acid and resveratrol can improve steroid production from TM3 Leydig cells following exposure with 250 μg/mL of extract for 48 h [171]. In one of our latest research, we’ve shown that gigantol (Figure 4), a bibenzyl compound isolated from orchids, can improve progesterone production in MA-10 Leydig cells by increasing the expression of genes involved in the biosynthesis of cholesterol and its import into mitochondria [8]. Thus, gigantol and resveratrol are phenolic compounds with great potential for preventing age-related declines in androgen production in men.

## 5. Other Actions of Natural Antioxidants

### 5.1. Effects of Natural Antioxidants on the Hypothalamus

Natural polyphenolic compounds have been studied for their potential effects on the hypothalamus, a regulatory center for various physiological processes. Potential effects of polyphenolic compounds on the hypothalamus include neuroprotection, hormone regulation, energy balance, and neurogenesis. Polyphenolic compounds, such as flavonoids, have been shown to possess antioxidant and anti-inflammatory properties that may help protect neurons in the hypothalamus from oxidative stress and neuroinflammation [172], potentially preserving hypothalamic function during aging. Polyphenolic compounds also modulate hormone secretion from the hypothalamus, influencing the release of various hormones such as GnRH [173], which controls the release of reproductive hormones LH and FSH. Recent evidence suggests that polyphenolic compounds may promote neurogenesis in the hypothalamus [174], which could have implications for cognitive function and overall brain health. However, only genistein has been reported to directly increase GnRH release from the mouse hypothalamic cell line GT1-7, potentially affecting reproductive function [175]. Hence, the specific mechanisms by which polyphenolic compounds regulate GnRH secretion and their implications in the regulation of testosterone production in aging males require further investigation.

### 5.2. Effects of Natural Antioxidants on the Pituitary Gland

Besides the hypothalamus, natural polyphenolic compounds may influence hormone production and release from the pituitary gland. Polyphenolic compounds may influence different functions of the pituitary gland, such as LH and FSH secretion and stress response, having effects on testicular androgen production. These compounds may also influence the pulsatile release of GnRH from the hypothalamus or directly affect pituitary gonadotroph cells.

Some polyphenolic compounds have been studied for their potential inhibitory effects on the pituitary gland-adrenal axis [176,177], which regulates the body’s response to stress and may influence testicular androgen production. These compounds may reduce the secretion of ACTH from the pituitary gland, leading to decreased cortisol release from the adrenal glands, thereby having an anti-stress effect. Indeed, polyphenol-rich dark chocolate has been reported to lower salivary cortisol in adults [178]. Cortisol may decrease cAMP-induced *Star* expression in Leydig cells as reported using the synthetic glucocorticoid dexamethasone [179]. However, the effects of polyphenols in reducing ACTH and cortisol levels on androgen production in the testis will require further study to be confirmed and may be the result of multiple actions of these natural antioxidants.

Several specific polyphenolic compounds have been investigated for their potential effects on LH release from the pituitary gland. EGCG and green tea extract have been reported to inhibit LH release from the pituitary gland [180], potentially influencing reproductive function in males. Although the LH pulses were decreased by intravenous administration of the phytoestrogen coumestrol, genistein had no effect on LH release from female rats [181]. However, the effects of genistein on LH secretion may vary depending on treatment conditions such as dose, duration of exposure, and sex of the animals. While some studies suggest that polyphenolic compounds influence LH release from the pituitary gland, further research is needed to fully understand their mechanisms of action and overall effects, as well as the contribution to prevention of male late-onset hypogonadism.

### 5.3. Influences of Natural Antioxidants on Liver Function and Testosterone Bioavailability

Liver function is very important for testosterone bioavailability, as steroid transport proteins such as albumin and SHBG are synthesized by the liver and released into the bloodstream. Natural antioxidants, such as polyphenolic compounds, have been studied for their potential influences on liver function and testosterone bioavailability. Overall, natural antioxidants can positively influence liver function by reducing oxidative stress, inflammation, and liver damage. Resveratrol is a well-known polyphenol having hepatoprotective properties [182,183]. It has been shown to reduce oxidative stress [184], inflammation [182], and liver damage [185,186]. Quercetin also has antioxidant and anti-inflammatory properties that benefit liver health [187]. It has been found to protect against liver damage and inflammation induced by toxins and alcohol [188,189,190]. EGCG also reduces liver inflammation, oxidative stress, and fat accumulation, potentially preventing liver diseases such as non-alcoholic fatty liver disease (NAFLD) [191,192]. Curcumin has been shown to protect the liver from damage caused by toxins, drugs, and various liver diseases [193,194]. Apigenin also exhibits hepatoprotective effects by reducing inflammation and oxidative stress in the liver [195,196].

Natural polyphenolic compounds may also influence testosterone bioavailability by regulating the liver’s production of SHBG and albumin. Indeed, the plasma levels of these transport proteins interacting with steroid hormones, including androgens, influence total and free testosterone plasma concentrations. It is particularly the free form of testosterone that is responsible for its physiological actions. Age and low protein intake are major determinants of elevated SHBG serum levels and decreased testosterone bioavailability in older men [197]. Thus, more efficient production of these transport proteins by the liver in the presence of natural polyphenolic compounds could limit the symptoms of age-related male hypogonadism. However, more research is needed to confirm a direct inhibitory action of polyphenolic compounds on SHBG and albumin synthesis by the liver.

## 6. Conclusions

**Figure 5:**
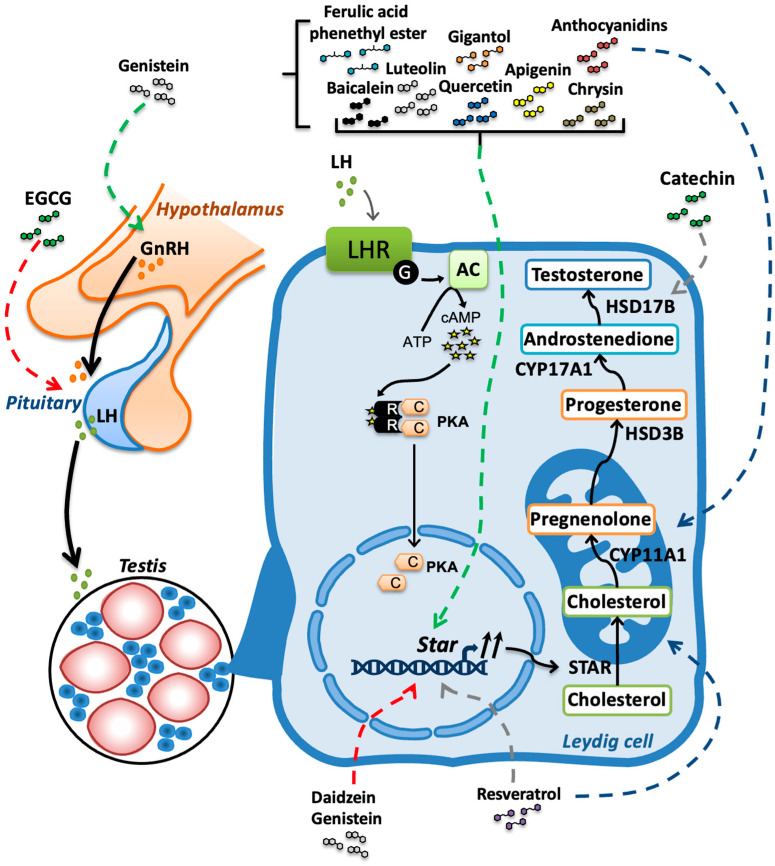
Summary of the mechanisms of action of natural polyphenolic compounds on testosterone production from Leydig cells of the testis. Flavonoids, isoflavonoids, resveratrol and gigantol mainly regulate steroidogenesis through the modulation of the expression of the *Star* gene. Dotted lines represent unknown intermediate steps in the regulatory mechanisms. Green arrows correspond to stimulation, red arrows to inhibition, and gray arrows to stimulation or inhibition, depending on context. The blue arrows correspond to improvement of mitochondrial function.

Importantly, plasma levels of natural polyphenolic compounds in the low micromolar range can be achieved with a high-quality diet consisting mainly of fruits and vegetables and are adequate for optimal testicular Leydig cells’ functions. Based on the research literature and our data, flavonoids with a 5,7-dihydroxychromen-4-one backbone have been observed to activate *Star* expression, leading to improved androgen synthesis by Leydig cells from the testis (Figure 5). While many polyphenols have activating or inhibitory actions on androgen biosynthesis, the combined exposure to natural polyphenolic compounds and their potential synergistic effects on steroidogenesis should be considered.

## Figures and Tables

**Table 1:** Summary of the important functions of testosterone in males.

Function	Description	References
Development of male reproductive tissues	Promotes the development of the testes, of epididymides and seminal vesicles, of the prostate, as well as of the penis and scrotum	[43,44,45,46,47]
Secondary sexual characteristics	Responsible for features such as increased muscle and bone mass, deepening of the voice, and growth of body hair	[48,49,50,51]
Adult male fertility	Critical for the initiation and maintenance of spermatogenesis	[52]
	Involved in the feedback regulation of pituitary gonadotropin production and secretion	[53]
Muscle mass and strength	Enhances muscle growth, increases protein synthesis, and improves physical strength	[48,54]
Bone growth and density	Increases bone density and helps maintaining bone health	[49,55]
	Enhances bone growth during puberty and cessation of growth of long bones at the end of puberty	[56,57]
Red blood cell production	Stimulates the production of red blood cells in the bone marrow	[58]
Mood and mental health	Affects mood, energy levels, and overall sense of well-being; low levels can lead to depression and fatigue	[59,60,61,62]
Libido and sexual function	Plays a critical role in sex drive and erectile function	[63,64]
Fat distribution	Influences the distribution of body fat, often leading to a reduction in fat mass	[65]
Cognitive function	Contributes to cognitive functions such as memory and spatial abilities	[38,39]
Hair growth	Promotes hair growth on the face and body, while potentially contributing to scalp hair loss	[51]

**Table 2:** Lipophilicity of flavonoids basic skeleton.

Basic Skeleton	Lipophilicity (Log *p*) *
Flavanoids	3.47
Flavones/Isoflavones	3.18
Flavonols	2.84
Flavanones	3.18
(+)-Catechin (R = H)	0.58
Anthocyanidins	2.22

* SwissADME [69].
